# Supplementary material for: Are carbon emissions trading and green financial instruments synergistic? -Comprehensive quantitative research based on content analysis
Source: PLoS One. 2024 Mar 7;19(3):e0298601. doi: 10.1371/journal.pone.0298601 (PMC10919601; doi:10.1371/journal.pone.0298601)
Supplement: S1 Data — (ZIP) [file pone.0298601.s001.zip › Data/地级市Z_F工作报告.zip!/║■─╧/│ñ╔│╩╨/└┤╫╘╣1⁄2╓┌║┼í╛┬φ┐╦╩2╛▌═°í┐_2013.docx]

各位代表：

　　现在，我代表市人民政府向大会报告工作，请予审议，并请各位政协委员和其他列席人员提出意见。

　　一、过去五年的工作回顾

　　过去五年，在市委的坚强领导下，我们以科学发展观为统领，抢抓中部崛起和两型社会建设的战略机遇，科学应对国际金融危机的严峻挑战，成功抗击历史罕见的雨雪冰冻灾害，推进创业之都、宜居城市、幸福家园建设，圆满地完成了市第十三届人民代表大会第一次会议以来确定的各项目标任务。

　　——综合实力大幅提升。2012年，全市地区生产总值达6510亿元（预计数，下同），年均增长14.6%，经济总量居省会城市第7位，五年前进了5位；人均地区生产总值达91400元（约14600美元），居省会城市第3位；辖内财政总收入达1509 亿元，其中地方财政总收入达813亿元，年均增长24.3%；完成固定资产投资4120亿元，年均增长25.8%。科技竞争力居全国省会城市第6位，被评为全国科技进步示范城市、国家知识产权示范城市；驰名商标总数稳居中西部首位；城市综合竞争力由第24位跃升到第10位，进入全国十强、全球十快。

　　——基础设施建设成效显著。营盘路隧道建成通车，福元路大桥竣工通车，过江交通能力大幅提高；湘府路大桥实现合龙，南湖路隧道单洞贯通，过江通道将达10条；株树桥引水工程建成，开辟了城市第二水源；湘江综合枢纽蓄水通航，枯水期缺水成为历史，山水洲城更加美丽；黄花机场新航站楼投入营运，进入全国十大机场行列；高铁南站、新火车北站投入营运，地铁2号线实现轨通，1号线加快推进，为长远发展提供有力支撑。

　　——农村面貌发生深刻变化。农村公路通达、电网扩容、安全饮水、环境整治、校舍改造五大工程全面推进，新改建农村公路9140公里，村级道路全部硬化；新改建低压线路近万公里；解决100万农村人口饮水安全问题；加固及重建、新建校舍25万平方米，校舍安全工程全面完成；农村垃圾收集处理实现全覆盖，成为全国农村生态环保示范城市，初步展现山清水美的新面貌。

　　——两型综合配套改革大力推进。推行阶梯水价、电价、气价和项目能评制度，建立环境资源交易平台和运用经济杠杆推动生态环保新机制，建立农村土地流转交易市场，创新人口户籍、行政管理、投资融资体制，成为全国首批节能减排财政政策综合示范试点城市，先导区获批全国生态文明建设试点区。

　　——人民群众生活持续改善。城乡居民人均可支配收入分别达30400元和15000元，稳居中部省会城市首位；五年新增城镇就业58.8万人，零就业家庭保持动态清零；取消长浏、浏大、长湘、宁横公路等政府还贷公路收费站，

　　普通公路全部免费通行；实现城乡义务教育“一费制”全免，城区公园和公共文化设施免费开放，暑期免费为中小学生提供游泳场所；荣获全国最具幸福感城市终身荣誉。

　　——政府管理服务全面加强。全面推进政府精细化管理，政府投资管理实行从项目立项、预算控制、工程建设到竣工决算全过程监管，通过优化设计核减投资47亿元，投资审计审减工程造价99.4亿元；建立公共交易平台，规范招标投标管理和国有资产处置；成立土地管理委员会，严格土地管理，率先全国实行土地出让网上招拍挂，确保土地出让公开、公平和公正；深化行政审批流程再造，市级行政审批项目全部实现在线办理，行政审批效率提升30%以上。

　　五年来，我们突出抓了以下几个方面的工作：

　　一是致力促进经济又好又快、率先发展。推进创业之都建设，鼓励支持创业富民，新增创业主体达20万户，非公有制经济占地区生产总值和从业人员总数比重均达60%以上，成为全国创业先进城市。发挥人才第一资源作用，引进储备优秀青年人才，引进国际高端人才101名、团队17个。推进新型工业化，实施质量振兴和标准化战略，加快优势产业和战略性新兴产业发展，工业总产值突破8000亿元，长沙高新区、长沙经开区成为千亿级园区，宁乡经开区、浏阳生物医药园晋升国家级园区。加快现代服务业发展，天心文化产业园成为国家文化产业示范园，文化对经济的贡献率居省会城市第三位，旅游总收入居省会城市第七位；社会消费品零售总额达2510亿元，年均增长19.2%；连续六年举办科交会，签订合作项目1328个，签约金额1139.5亿元；成功举办环湘江自行车赛、中博会、农博会等重大节会，成为国家现代服务业综合试点城市和服务外包示范城市。加大对外开放，金霞保税物流中心、长沙国际邮件监管中心投入运营，新增世界500强企业14家，引进外资银行5家；累计完成进出口总额314.3亿美元，年均增长13.4%；累计实际利用外资116.5亿美元，年均增长13.4%；新增友好城市或友好合作城市10个，荣获国际友好城市交流合作奖。被评为中国（大陆）国际形象最佳城市、福布斯中国大陆最佳商业城市。

　　二是致力精心规划、精致建设、精细管理城市。以规划引导城市发展，开展《城市总体规划》修编，望城撤县改区，城区面积扩大一倍；实施《城市色彩规划》，加强城市绿线管理，强化重要区域、节点设计，加强单体设计和外立面审查，城市色彩更加简洁、素雅。实施“沿江建设、跨江发展”，加快重点片区开发，相继建设一批城市综合体，形成湘江两岸互动共进新格局。树立全寿命周期成本最低理念，推进精品工程建设；完成中山西路、东牌楼等棚户区改造近500万平方米，高升村、红星村等城中村改造试点顺利推进；大浏、浏醴、长湘等高速建成通车；湘江生态经济景观带防洪道路工程竣工。连续四年实施交通疏导工程，成为全国畅通工程模范管理城市；实施黄花机场至市区沿线和城乡结合部环境综合整治，提质改造农贸市场174个，建成社区公园80个、特色景观街21条；生态动物园搬迁开园，岳麓山—橘子洲景区成为国家5A级旅游景区，沩山入选国家级风景名胜区；森林覆盖率达53.4%，建成区绿化率达42.2%，空气质量优良率保持在90%以上。被评为国家园林城市、国家绿化模范城市，联合国授予中国区环境规划示范城市。锲而不舍地推进文明创建，成功夺得全国文明城市桂冠。

　　三是致力加快城乡一体化发展。加快城镇化步伐，启动基础设施项目1920个，完成投资近400亿元，城镇化率达68.5%。开展农村土地综合整治，莲花镇、开慧镇、关山村、光明村等成为新农村建设示范。大力发展花卉苗木、有机茶叶、高产油茶、优质水稻、特色水产五大优势产业，加强标准化规模养殖和专业蔬菜基地建设，粮食生产实现“九连增”，农林牧渔业增加值实现翻番。推进农业科技创新，成为国家农村农业信息化示范市。建立新型农业经营体系，农业产业化龙头企业达307家，农民专业合作组织带动30多万农户增收。推进城乡公共服务均等化，率先全国实现城乡居民医疗保险并轨，城乡养老保险制度全覆盖，五年四次提高城乡低保标准。改建农村和国有林场危房2.1万户。所辖县（市）在全国县域经济基本竞争力百强中排名大幅前移，长沙县居全国中小城市综合实力第13位、中西部地区首位。

　　四是致力推进两型社会建设。先导区“六纵八横”交通框架全面形成，天马山、凤凰山、桃子湖提质改造完成，洋湖湿地公园一期开园，坪塘老工业基地污染企业全面退出，梅溪湖新城获批全国首批绿色生态示范城区，先导区建设为长沙发展增添新动力。坚持生态优先，实施两轮环保三年行动计划，城区规模养殖基本退出，完成铬盐厂42万吨铬渣解毒处理，城市污水处理率达95%，城市垃圾无害化处理率达100%，成为全国餐厨垃圾无害化处理试点城市。坚持资源节约集约利用，宾馆、酒店停止免费提供一次性用品并实施节电综合改造，推进绿色建筑试点示范，全面完成节能减排目标任务，单位地区生产总值综合能耗居中部省会城市先进水平。探索推广住宅人车分离、农民高层安置、高层工业厂房等节约集约用地模式。发挥城投集团、先导控股、轨道集团投融资作用，探索企业债券、集合委托贷款、信托等多元融资方式。长沙两型社会建设为全国生态文明建设探索了新路子。

　　五是致力改善民生和加强社会建设。坚持将公共预算支出的70%以上用于民生保障，全面完成为民办实事工程。推进教育均衡发展，合格学校建设全面完成，建成一批高品质学校，职教基地投入使用，双轨制学校改为公办，新增公办幼儿园160所，高等教育质量不断提高。实施基层医疗机构综合改革和基本药物制度，开展公立医院改革试点，基本形成城区“15分钟医疗服务圈”。初步形成覆盖城区的居家养老服务体系。市老干部活动中心建成开放。推进保障性安居工程和经适房货币化改革，城市居民人均住房面积达33.1平方米。实施农家书屋、广播电视户户通工程，推动国家公共文化服务示范区创建，铜官窑遗址公园建成开园。加强体育设施建设，开展全民健身运动，成功举办第十届全国中学生运动会。推进全国社会管理创新综合试点，深入开展治安、治差、治堵、治难、治脏专项活动，加快“天网工程”建设，生产安全、消防安全、应急管理、特殊人群管理服务不断加强。完善群众工作体系，建立矛盾纠纷化解机制，解决一批信访遗留问题。加强食品安全工作，实施“放心猪肉、放心蔬菜、放心作坊、放心食堂”工程，食品检测合格率达98%，连续十年无源发性药品安全质量事故。推进流动人口基本公共服务均等化，成为全国人口计生综合改革示范市。加强价格监管，居民消费价格指数低于全国省会城市平均水平。民族政策全面落实，宗教形成和顺局面。顺利完成经济普查、人口普查、“五五”普法和第八次村委会及社区换届。

　　六是致力加强政府自身建设。坚持科学民主决策，重大决策广泛征询意见，实行市民代表列席政府常务会议和市长办公会制度。坚持依法行政，开展重大行政决策和地方立法听证，率先全国出台首部政府法制工作规定。完成政府机构改革，便民服务中心实现全覆盖。加强政务公开和政务服务，取消行政事业性收费208项，群众办事更加方便。加强政府廉政建设，发挥行政监察作用，坚决治理不正之风。坚持厉行节约，突出抓好资金、资产、资源监管，推行财政绩效评价，深化国库集中支付和政府采购采管分离改革，进一步规范“三公经费”支出。国资监管实现全覆盖，国企改革任务基本完成。严格基本建设程序，强化政府重大合同审查、可研评审、招投标和规划管理，重大方案反复论证，政府投资管理不断规范。自觉接受人大工作监督和法律监督，主动接受政协民主监督，积极开展民主协商，加强与各民主党派、工商联对口联系。五年来共办理人大代表议案4件；办理建议、批评、意见1446件，满意率达99.8%；办理政协提案1920件，满意率达99.5%。

　　国防、人防和后备力量建设实现新发展，驻长部队在抢险救灾等急难险重任务中发挥了重要作用，军政军民关系和谐融洽，荣获全国双拥模范城“五连冠”。工会、共青团、妇联、科协、文联、侨联、社科联、残联、贸促会、红十字会、关工委、老龄委等为长沙发展作出新贡献。国家安全、对台、档案、机关事务管理、市志、供销合作、公积金管理、海事、气象、地震、移民开发等工作取得新成绩。

　　各位代表，过去五年是长沙经济社会发展最快、城乡面貌变化最大、人民群众受益最多的时期之一，我们站在了更高的起点上。这些成绩的取得，是省委、省政府和市委正确领导的结果，是市人大、市政协和人大代表、政协委员监督支持的结果，离不开历任老领导、老同志的关心帮助，是全市人民团结奋斗、苦干实干的结果。我们始终心存感激、充满敬意！在此，我谨代表市人民政府，向全市各族人民，向驻长部队、武警官兵和政法干警，向各民主党派、工商联、人民团体和各界人士，向所有关心支持长沙发展的国内外朋友，表示衷心的感谢！

　　过去五年，我们在实践中积累了一些宝贵的经验：一是必须坚持科学发展，率先发展。始终把发展作为第一要务，凝心聚力抓经济，一心一意谋发展，尊重经济规律，重视基础工作，强化发展质量，调整产业结构，优先实施拉动作用大、群众受益大的重大基础设施、产业发展和民生工程项目，实现又好又快、率先发展。二是必须坚持依靠群众，服务群众。尊重人民群众的主体地位，坚持把群众利益放在首位，把改善民生作为工作的出发点和落脚点，以信赖群众、依靠群众推动发展，以尊重群众、服务群众赢得理解和支持，建设长沙人自豪、外地人向往的幸福家园。三是必须坚持国际视野，理念创新。坚持以国内领先、国际先进水平为坐标，以国际视野指导发展实践，用现代理念推动政府工作，冲破惯性思维，创新工作理念，大胆借鉴国际国内先进经验，长沙更加开放、更加自信、充满活力、富有魅力。四是必须坚持勇于负责，敢于担当。牢固树立宗旨意识，牢记责任和使命，坚持公共利益至上、群众利益至上，凡是有利于长沙发展、有利于人民群众的事，就埋头苦干、大胆推进，不回避矛盾，不畏惧困难，不计较个人得失，对历史负责、对人民负责。

　　在看到成绩的同时，我们也清醒地看到发展中还存在不少的不足和问题：产业结构尚未形成多点支撑，与人民生活密切相关的终端产品偏少；城市建设管理还存在薄弱环节，区域之间、点面之间发展不平衡的矛盾比较突出；新农村建设任务依然繁重，基础设施仍不完善；上学难、看病贵等问题解决得不够好，特别是低收入群体生活与全面小康的目标还有较大差距；少数政府机关工作人员服务意识、办事效率与群众期待还有一定差距，发展环境有待进一步优化。对此，我们必须高度重视，采取有效措施认真加以解决。

　　二、未来五年的发展建议

　　各位代表，未来五年，长沙处在可以大有作为的重要战略机遇期，也是全面完成“十二五”规划目标任务、率先建成全面小康社会的关键时期。我们必须认真贯彻落实党的十八大精神，按照市第十二次党代会确定的“城市国际化、产业高端化、城乡一体化、发展两型化、管理法治化，率先建成两型城市和实现全面小康”的战略目标，着力转变发展方式、推动改革创新、提升城市品质、推进城乡一体、创新社会管理、保障改善民生，努力建设更加富裕、秀美、文明、幸福的大长沙。

　　——建设经济繁荣、活力迸发的现代中心城市。实现由“追赶型”发展向“引领型”发展转变，创新创业日趋活跃，要素资源竞相汇聚，成为全国重要的产业基地。地区生产总值迈上万亿台阶，财政收入实现翻番，综合实力挺进省会城市“一方阵”，现代化建设走在全国城市前列。

　　——建设两型引领、环境优美的生态宜居之都。生态文明理念深入人心，资源利用更加集约节约，流域水质明显改善，大气环境更加洁净，城乡生态更加优美，城市功能更加完善，居民出行更加便捷，山水洲城风貌凸显，成为天蓝、山青、水绿，人与自然和谐相处的全国生态文明示范城市。

　　——建设开放包容、魅力独特的国际文化名城。对外开放全面深化，国际交流日益广泛，文化内涵更加丰富，国际影响力显著增强，成为历史文化与现代文化交相辉映、湖湘文化与国际文化互动交融、文化事业与文化产业比翼齐飞的文化名城。

　　——建设生活富足、社会公平的和谐幸福家园。城乡居民充分就业，居民收入与经济总量同步增长，社会保障全面加强，民主法治不断健全，群众均等享受基本公共服务，社会更加公平正义、安定和谐，人民生活更加殷实、更有尊严，率先全面建成小康社会。

　　主要预期目标是：到2017年，地区生产总值达12000亿元，年均增长11%；地方财政总收入达1600亿元，年均增长15%；完成固定资产投资8300亿元，年均增长15%；实现社会消费品零售总额5000亿元，年均增长15%；城乡居民人均可支配收入分别达53000元和29000元，年均增长12%和14%；城镇登记失业率控制在4%以内；单位地区生产总值综合能耗累计降低15%，完成省政府下达主要污染物排放总量减排指标；人口自然增长率控制在7％。以内；居民消费价格指数在省会城市平均水平以下。

　　围绕上述目标任务，建议重点抓好以下五个方面的工作：

　　（一）加快转变发展方式，提升经济发展质量

　　打造先进制造业中心。坚持以科技创新为主抓手，以工业园区为主战场，推动产业集群化、高端化发展。保持工程机械产业优势，支持烟花鞭炮、食品、服装等传统产业创新发展，将汽车制造、电子信息、集成建筑、新材料培育成为新的千亿产业，形成多点支撑的工业发展格局。实施知识产权战略，提高质量标准意识，突出引进创新创业人才，实施国家科技重大专项，加快科技成果转化，完善科技创新体系。创新招商方式，注重引大引强，配套基础产业，助推产业升级。着眼差异化发展，避免同质化竞争，促进产城融合，将宁乡、浏阳、望城、金霞经开区打造成为新的千亿园区，长沙高新区、长沙经开区工业总产值分别迈上3000亿元台阶，实现工业总产值、规模工业增加值倍增。

　　打造现代服务业中心。坚持以激活消费扩大内需，提升现代服务业发展水平。积极发展现代物流、技术咨询、工业设计等生产性服务业，建设高端企业总部聚集区。突出发展商贸流通业，合理布局大型商品批发市场、商业网点和仓储物流基地，建成国际会展中心和一批城市综合体。繁荣中央商务区，推动高端消费市场发展。提质改造传统服务业和传统商业中心，加大湘菜振兴扶持力度。加快旅游强市建设，打造“快乐长沙”旅游品牌，建设世界旅游目的地。坚持对内与对外开放并举、出口与进口贸易并重，加快发展电子商务和网上购物，推动内外贸易快速发展。实现社会消费品零售总额、旅游总收入翻番，建成中部现代服务业中心。

　　打造文化创意中心。推动文化产业与相关产业融合发展，把文化产业培育成为经济发展的新引擎。加快建设天心文化产业园、中南国家数字出版基地、长沙（国家）广告产业园，规划建设一批省市重点文化创意园区和文化产业项目，提高文化产业规模化、集约化、专业化水平。支持长沙广电发展壮大，打造知名文化企业，培育国际文化传播品牌，加强国际文化交流，形成独具长沙特色的文化符号，文化产业增加值占地区生产总值10%左右。

　　打造区域金融中心。科学规划金融产业布局，加快芙蓉路和滨水金融生态区建设，引进国内外金融机构区域总部、金融后台服务中心10家以上。发展壮大地方金融机构和投融资平台，支持符合条件的企业上市，规划建设金融市场，积极发展民营中小银行和小微企业融资综合服务中心，直接融资超过25%，地方金融资产占金融机构总资产35%以上。健全金融体系，优化金融生态，完善信用体系，扶持创业投资，规范民间借贷行为，基本形成多层次资本市场，金融业增加值占地区生产总值5%左右。

　　（二）加强规划建设管理，提升城市宜居品质

　　建设精美长沙。推进“沿江建设、跨江发展”，加快大河西先导区建设，拓展城市发展空间。重点完善省府新区、星沙新城、麓谷新城，加快发展隆平新区、黎托新城、滨江新城、金霞新城、望城滨水新城、宁乡金洲新区，启动建设黄兴新区、空港新区、雷锋新区、黄金新区，重点建设南湖、洋湖、梅溪湖、大王山片区，规划开发苏托垸、解放垸、雅塘村片区。精心雕琢重点街区、重要街道和城市节点，高度重视工程质量，打造百年精品工程，形成起伏有序的城市天际线。推进精细化管理维护，加快城中村、棚户区、老旧社区提质改造，激活房产二级市场，促进房地产市场平稳健康发展。完善湘江生态经济带，打造“浏阳河九道湾”品牌，规划改造捞刀河、沩水河、靳江河、圭塘河风光带，构建魅力彰显的城市景观带。形成以湘江为轴、大河西先导区与河东主城区互动发展、山水洲城自然融合的宜居都市。

　　建设畅通长沙。发展综合交通，建设全国交通枢纽城市。完成黄花机场二期改扩建，建成全国枢纽机场和重要国际航空港。支持城际铁路、沪昆高铁、渝厦高铁建设，建成现代高铁枢纽。加快湘江航道和霞凝港三期建设，建成湘江综合枢纽。完善高速公路路网，实现乡镇驻地与高速公路直连。研究建设纵贯南北、横贯东西的快速通道，完善主城区南部路网，推进湘江大道、潇湘大道南拓北延、枫林西路西延，建设芙蓉大道北延线、人民东路东延线。治理城区堵点，打通微循环。坚持公交优先、公益主导的发展体制，建成4条地铁线路，完成高铁南站、大河西、汽车南站等综合交通枢纽，全面建设公交都市。鼓励发展立体停车场，新建建筑配足停车位，清理恢复改变用途的停车场，着力解决停车难。完善智能交通诱导系统，加强交通宣传教育，培育文明交通行为。

　　建设数字长沙。加快“三网融合”，推广运用信息网络技术，建设中部重要的通信枢纽，建成覆盖全市、统一集中的数据库及管理中心，健全信息安全保障体系。发展云计算产业，重点支持国家超级计算长沙中心建设，加强互联网数据、生产运营、呼叫、容灾备份等信息服务平台建设，培育在线支付等新型业态。推进政府上网工程，打造数字城管，建成数字档案馆。统筹建设地下空间和地理信息平台，构建全市统一的“市民卡”系统，促进社会事业和公共服务信息化。实现城区光纤入户、农村光纤到行政村，中心城区、县城和重点乡镇基本实现无线宽带全覆盖。

　　建设生态长沙。严格绿线管理，保护城乡生态环境，巩固发展城市林业生态圈。实施重大生态修复工程，加快傅家洲等江心洲保护性开发，全面提质国家森林公园。新建一批湿地公园、城市综合性公园、社区公园和城市绿道，构建“15分钟绿色生活圈”。落实阶梯水价、电价、气价制度，推进节地、节能、节水、节材和清洁生产、低碳生产，积极发展应用新能源与可再生能源，加快非两型产业退出，全面完成节能减排目标。持续推进环保三年行动计划，加大江河流域综合治理，加强大气污染联防联控，推进农村环境综合整治，开展生态文明示范创建。推进垃圾减量化、资源化、无害化处理，推行居民垃圾分类收集和深化处理。实现环境质量、生态指数、单位地区生产总值能耗水平居国内先进水平，建设“绿带”环城、“绿道”满城、“绿点”遍城的绿色城市。

　　（三）加快城乡一体化发展，提升农村发展水平

　　完善城镇总体布局。坚持集聚生产要素、集中农村人口、集成城市功能，积极稳妥、有序推进城镇化。加快浏阳“一线三城”建设，加速宁乡东部新城建设，把浏阳、宁乡建成中等规模卫星城市；灰汤、大瑶、永安、铜官、暮云等建成10万人以上的小城市；金井、镇头、流沙河、沿溪、花明楼等建成5万人以上的中心镇；开慧、乔口、沩山、大围山、莲花等建成风貌独具的特色镇；提质改造中和、靖港等小城镇；支持边远乡镇加快发展。结合乡村山水风貌，科学规划农民住宅，打造依山傍水、相对集中、错落有致、富有湖湘特色的民居村落和田园美景。

　　完善农村基础设施。坚持城乡基础设施同步规划、同步建设、有序对接，增强农村综合功能。加强农田水利建设，完成病险水库和病险水闸除险保安，整修加固中小河道，基本解决农村安全饮水问题。完成农村电网改造。加强农村消防基础设施建设。实施农村公路安保提质工程，按标准硬化通村公路，加强农村公路养护。推进农村垃圾分类收集处理，科学建设乡镇污水处理厂。消除马路市场，提质改造一批农贸市场。全面改善农村生产生活条件。

　　完善现代农业体系。坚持用工业化理念、市场化要求谋划农业发展，推进现代农业推广示范区建设，发展产业集中区，建设优质农业产业带，扶持市级以上农业园区发展，大力发展现代休闲观光农业，提升望城全国休闲农业示范区（县）影响力，构建近郊都市农业经济圈、中郊特色优势农业经济圈和远郊生态农业经济圈。推动农村流通现代化，着力破解农产品“买难卖难”问题。发展农产品精深加工，实现农产品加工业销售收入翻番。

　　完善农村公共服务。落实强农惠农富农政策，提高村级组织发展能力。加强农民创业就业服务，保持农民收入持续增长。依法推进征地拆迁，稳妥解决被征地农民安置、就业、社会保障问题。加强农村公共文化服务，建设一批集中居住区文化广场。完成乡镇卫生院、村卫生室标准化建设，完善小城市、中心镇医疗卫生服务。积极发展村镇银行，开展农业保险试点，完善农村金融服务体系。逐步推进“村改社区”，建设农村社区一站式服务平台。有序引导农业转移人口市民化，实现城乡基本公共服务均等化。

　　（四）加快民生社会事业发展，提升人民幸福指数

　　提高教育文化水平。坚持教育优先发展，教育资源注重向农村和边远地区倾斜，加强素质教育，促进教育公平；鼓励发展学前教育，均衡发展义务教育，积极发展职业教育和成人教育，推进高中教育、高等教育内涵式发展，规范和引导社会力量办学，重视教师队伍建设，办好人民满意的教育。加强不同层次人才的培养、引进和使用，满足各行业人才需求。实施文化惠民工程，建设重大公共文化项目，提质改造太平老街等历史文化街区，促进物质文化遗产和非物质文化遗产的保护、传承与开发利用，创建国家公共文化服务示范区。加强社会主义核心价值体系建设，全面提升公民道德素质，深入开展文明创建活动，推动学雷锋活动常态化，建设更高水平的文明城市。

　　提高社会保障水平。实施就业优先战略和更加积极的就业政策，实现更高质量的就业。加强保障性住房建设与管理，逐步解决困难群众住房问题。坚持全覆盖、保基本、多层次、可持续方针，稳步推进社会保险市级统筹，建成覆盖城乡的社会保障体系。做好优抚安置工作，完善社会救助体系，健全社会福利制度，支持发展慈善事业，促进残疾人事业发展。稳步提高城乡低保标准，推行农村“五保户”集中供养，照顾安排好困难群体和弱势群体基本生活。加强“菜篮子”工程建设，保持物价总体平稳。加强民族团结，促进宗教和睦。让人民群众生活更有保障、更有品质。

　　提高医疗健康水平。实施“健康长沙行动计划”，合理布局医疗卫生资源，规划建设省府新区、黎托新城、滨水新城三甲医院；推进医药卫生体制和公立医院改革，加强基层医院建设，引导、规范社会力量办医，率先实现人人享有基本医疗服务。深入开展爱国卫生运动，创建全国卫生城市。统筹抓好人口与计划生育工作，提高出生人口素质。加强食品药品生产经营全程监管，推进食品安全城市建设。加强体育设施和青少年活动场地建设，广泛开展全民健身运动，促进群众体育与竞技体育协调发展。持续推进“惠老工程”，完善社会化养老服务体系，人均预期寿命提高到80岁以上。

　　提高社会管理水平。加强和创新社会管理，促进社会管理法治化、网格化、长效化。加强法制教育，完成“六五普法”。加强群众工作，创新信访制度，引导群众依法表达诉求，发挥人民团体、社会组织作用，构建“大调解”格局，维护社会稳定。健全立体化社会治安防控体系，完善流动人口与特殊人群管理服务，依法防范和惩治违法犯罪活动，提高人民群众安全感。加强企业依法治理，维护良好的经济法治环境。加强未成年人保护，保障妇女儿童合法权益。强化公共安全体系和企业安全生产基础建设，加强气象、地质、地震灾害防御工作，完善突发事件应急处置救援机制。增强社区服务功能，提升社区物业管理水平。提高国防动员和后备力量建设质量，发挥部队支持地方发展的积极作用，发展人民防空事业，巩固和发展军政军民团结，争创全国双拥模范城“六连冠”。

　　（五）加强和改进政府管理，提升政府服务效能

　　牢固树立公仆意识。每一个政府工作人员都是人民的公仆，任何时候都要把群众利益放在首位。高度重视群众切身感受，时常换位思考，理解群众生活的不易、办事的困难、创业的艰辛，设身处地为群众排忧解难，着力解决人民群众最关心、最迫切、最现实的切身利益问题，满腔热忱地服务群众、服务企业、服务基层，不负人民的信任与重托。

　　大力弘扬实干精神。空谈误国，实干兴邦。再美好的规划，再宏伟的蓝图，归根到底靠实干才能实现，所有政府工作人员都要做实干的表率。要坚决整治慵懒、散漫、奢侈等不良风气，坚决克服形式主义、官僚主义，杜绝任何形式的形象工程、政绩工程，让实干成为政府工作主旋律，用实干赢得群众信任，用实绩回报群众支持。

　　全面提高服务水平。加强公务员队伍建设，开展专业能力和素质培训，提高政府工作人员的服务意识、成本意识和专业素养，提升政府整体效能和系统解决问题的能力。切实改进会风、文风、作风，减少迎来送往和各种庆典活动。优化行政审批流程，减少审批事项，坚决杜绝不作为、缓作为、乱作为的现象，坚决整顿不给好处不办事、给了好处乱办事的歪风，提高办事效率，改进服务质量，建设人民更加满意的政府。

　　努力建设法治政府。坚持依法行政，规范行政处罚自由裁量权，完善行政问责体系，做到每一项政府决策、每一项行政行为都符合宪法和法律的规定。完善行政决策程序，凡涉及群众切身利益的行政决策都充分听取群众意见。自觉接受人大的法律监督、工作监督，把政治协商、民主监督纳入决策程序，扩大民主党派、无党派人士、公民有序参与政府管理。强化行政执法监督，高度重视行政监察、审计监督和社会、舆论监督，让监督的阳光照亮权力运行的每一个角落。

　　切实加强廉政建设。建设清正廉洁的政府是人民群众的共同期盼。完善符合科学发展观的绩效考核体系，树立“重政绩、重公论”的用人导向。要坚持勤俭办一切事情，事事精打细算，处处严格把关，政府是受人民的委托管理城市、管理资金，管好、用好纳税人的每一分钱是我们的基本职责，没有任何理由铺张浪费。加强国有资产监管，构建科学有效的监管机制。加强对重点领域和关键岗位廉政风险防控，严格规范权力行使，加强经济责任审计，严肃查处违法违纪案件，树立干部清正、政府清廉、政治清明的良好形象。

　　各位代表！应对复杂多变的经济形势、完成繁重的工作任务、顺应人民对美好生活的向往，必须依靠高素质的人才、高品质的城市、高效率的政府，营造投资洼地，推动率先发展。要更加注重企业发展。企业是实体经济的基石。长沙的发展需要成千上万的企业，需要千千万万个懂经营、会管理、主动承担社会责任的企业家，要更加尊重、关爱企业家，注重发挥企业家才能，为企业家创业成长、创新发展营造公平竞争环境，满腔热情为外来投资者创业发展服务，积极支持本土企业发展壮大，促进实体经济有质量、有效益、可持续增长。要更加注重基层建设。基层是经济社会发展的依托。任何时候都必须把基层牢牢装在心中，加强基层基础工作，主动关心基层干部成长，营造舒心干事的良好环境，让基层成为建功立业的主战场。要特别关心农村基层建设，支持边远地区发展，始终把解决“三农”问题作为全部工作的重中之重，推进城乡一体化发展。要更加注重生态文明。青山绿水是大自然赐予长沙的宝贵财富。要格外珍惜长沙的山山水水，让自然优势与城乡建设有机结合，全面促进资源节约集约利用，打造两型社会建设的亮丽名片，让城乡环境更加优美，努力建设生产发展、生活富裕、生态良好的美丽长沙。要更加注重改善民生。改善民生是政府工作的根本任务。要鼓励和帮助每一个劳动者勤劳致富，通过加快发展做大“蛋糕”，让群众更多更公平地共享发展成果，决不因发展遇到困难而忽视民生保障，决不因收支矛盾加大而减少民生投入，以民生改善凝聚发展合力、推动经济增长。

　　三、2013年的工作重点

　　2013年是贯彻落实党的十八大精神的开局之年，也是新一届政府履职的第一年，要努力实现良好开局。全市经济社会发展主要预期目标为：地区生产总值增长12%以上，地方财政总收入增长15%，固定资产投资增长20%左右，社会消费品零售总额增长16.5%，城乡居民人均可支配收入均增长14%左右，城镇登记失业率控制在4%以内，单位地区生产总值综合能耗降低4%，完成省政府下达主要污染物排放总量减排指标，人口自然增长率控制在6.6％。以内；居民消费价格指数控制在103.5以内。为此，按照稳中求进的工作总基调，着力抓好以下工作：

　　（一）推进产业转型升级。加速推进新型工业化。围绕构建现代工业体系推进新型工业化，推动烟花、食品等传统产业提质发展，工程机械、汽车制造等优势产业集群发展，支持电子信息等科技型企业创新发展。突出重大产业项目支撑，加快新引进项目签约落地，促进在建重大产业项目达产达能。加快发展战略性新兴产业。按照先导性、支柱性要求，重点扶持高端制造、新材料、新能源产业，优先发展节能环保、新能源汽车、生物医药、电子信息和文化创意产业，延长优势产业链，发展终端产品，促进高端发展。提升现代服务业水平。大力发展电子商务，搭建第三方支付平台，建设中小商贸流通企业公共服务平台。启动长沙国际会展中心建设，加快商贸批发市场外迁。加速岳麓山、灰汤温泉、大围山、花明楼等旅游产业功能区开发，建设游客集散中心。扶持发展湘菜产业，促进传统服务业发展。强化产业发展支撑。合理布局园区产业，支持金霞经开区创建国家级现代物流园区，推进隆平、天心、雨花、岳麓等园区发展。强化人才支撑，健全科技创新体系。加快长沙金融生态区建设，发挥城投集团、先导控股、轨道集团平台作用，支持长沙银行、水业集团、粮食集团等符合条件的企业上市，破解企业融资难题。

　　（二）推进宜居城市建设。加强规划设计。启动新一轮城市规划调整研究，完成《城市总体规划》修改报批，加强望城与主城区规划对接，加强村镇规划编制。完善城市交通规划，加强历史文化街区、风貌区和交通枢纽等重点区域规划设计。完善基础设施。实现地铁2号线、石长铁路复线、湘府路大桥、南湖路隧道建成通车。加快地铁1号线、城际铁路、沪昆高铁、湘江综合枢纽、综合交通枢纽等项目建设，开工建设地铁3、4号线、京珠高速辅道、营盘路东延线、荷花路浏阳河隧道、铁路新北站二期等重大项目。提升城市品质。加快黎托新城、省府新城、滨江新城、朝正垸片区等开发建设。推进新一轮旧城改造，科学保护与开发、利用文化遗产。扩大“城中村”改造试点，加快黄兴北路、书院路、古道巷、长重等棚改步伐，启动经适房上市交易，促进房地产市场健康发展。实施地下管网、自来水户表改造，推进老旧厂区水电分离。统筹推进浏阳河风光带建设，提质改造晓园、白沙井等城市公园，新建社区公园20个，城区新增绿地300公顷、绿化达标小区100个。提高管理水平。推进公交都市建设，加快公交企业整合重组，加大公交站场建设管理力度，加强公交线路运营和票价管理，规范出租车运营管理，严厉打击非法营运。继续实施畅通工程，完善交通微循环。实施更高水平的精细化管理，推进城乡结合部环境综合整治，提升城区夜景亮化品质，开展社区环境整治，规范社区物业管理。健全文明创建长效机制，巩固文明创建成果，不断提升城市文明程度。

　　（三）推进城乡统筹发展。加快建设中心城镇。重点支持小城市、特色镇、中心镇建设，推进中心城镇与周边乡镇协调发展，加大对边远乡镇产业发展扶持。加快城乡基础设施对接，加速农村电网扩容改造，修建农村公路900公里，推进农村公路安保提质和危桥改造。解决30万农村人口安全饮水问题。加快发展现代农业。大力发展清洁种养技术，提高粮食生产能力，推进蔬菜基地建设，发展精品农业和林下经济，促进畜禽养殖产业化经营，保护发展宁乡花猪等地方特色品种。提高农业机械化水平，发展农产品加工业，加速都市农业、休闲农业发展，提升农业规模、质量和效益。加快农业经营体系建设。积极发展农村金融，科学布点村镇银行，大力发展农场、农业企业、专业合作社等现代农业组织，构建新型农业支付、流通经营体系。创新土地流转，治理抛荒行为。完善现代农产品配送体系，开展市内农副产品直销配送，启动农产品集配中心建设。加快完善农村公共服务。扩大农村低保、医保和养老保险覆盖面，加快农村危房改造步伐，启动新一轮农村敬老院改扩建。实施科技增收工程，加强农民技能素质培训，妥善解决被征地农民和农民工社会保障和就业问题。多途径壮大集体经济、增加农民收入。

　　（四）推进两型社会建设。加速先导区建设。加快滨江新城、梅溪湖片区、洋湖片区、大王山旅游度假区和望城滨水新城、宁乡东部新城建设，实现洋湖湿地公园、西湖文化园、桃花岭公园、梅岭公园开园。大力发展两型产业，集聚发展总部经济、金融服务、文化创意、商贸商务等新兴业态。推进重点领域改革。落实环境经济政策，健全资源环境管理体系，加强资源环保审计，加大绿色信贷、绿色技术支持力度，完善公众参与、环保自治机制。开展节能减排财政政策综合示范，推进土地管理改革，实施铬渣污染深度治理，推进企业合同能源管理。加大环境保护力度。推进新一轮“环保三年行动计划”，实施湘江综合枢纽库区清污工程，继续加大污水管网建设力度，加强湘江水体保护和浏阳河、捞刀河水污染治理。实行垃圾分类减量收集与处理，加强餐厨垃圾收集处理及综合利用。继续治理畜禽养殖污染，新建、续建乡镇污水处理厂12家。加快黄标车淘汰退出，启动PM2.5监测，空气质量持续改善。

　　（五）推进开放型经济发展。提升招商引资水平。发挥侨联组织、行业协会、商会和海外归国人员的积极作用，加大与世界500强企业、跨国公司、央企、上市公司的合作，围绕产业发展方向引进主机企业和配套企业。优化投资环境，提高合同履约率、资金到位率、项目开工率，增强招商引资实效。夯实对外开放平台。抓住发达经济体和先进地区产业转移机遇，主动承接战略产业转移。支持建设临空经济区、现代物流区，建设国家级综合保税区。完善部门协作机制，建好口岸联检机构，建设口岸电子信息平台，提高口岸通关能效。发展对外经贸合作。增强企业国际化经营能力，发展拥有自主知识产权的出口品牌，提升产品竞争力，积极参与国际竞争，提高国际市场占有份额。促进加工贸易转型升级，发展服务贸易，促进对外贸易平衡发展。

　　（六）推进民生事业改善。优先发展教育。合理配置教育资源，加强标准化学校建设，大力培养农村教师和优秀骨干教师，把优质教育办到群众家门口。保障外来务工人员子女平等接受义务教育，加强农村留守儿童服务，支持发展普惠性幼儿园，落实中等职业教育免学费政策。促进文化繁荣。完成“两馆一厅”建设，加快青少年宫、汉王陵和炭河里国家考古遗址公园建设。打造一批街道（乡镇）、社区（村）文化服务示范点，巩固农家书屋建设成果，继续实施送戏下乡等文化惠民工程，丰富人民群众文化生活。扶持重大文艺精品生产，支持金鹰文化城等重点产业项目发展，加强国际文化交流。　　提高保障能力。完善就业服务体系，创造更多公益性岗位帮扶困难群体就业，让群众更加体面地劳动，通过勤劳致富改善生活。加强城乡社会保障体系建设，提高社会保险覆盖面。合理规划养老设施布局，支持开展社区养老，提高老年人生活质量。建立农村五保、城乡低保标准自然增长机制，设立临时救助制度并向低收入家庭延伸。完善优抚、助残、救孤、济困等制度，发展慈善事业。加强和改进价格调控监管，努力保持“菜篮子”主要产品价格基本稳定。提升健康水平。积极开展群众体育活动，办好市第八届运动会。扩大公立医院改革试点，基本完成村（社区）标准化卫生室建设，让群众在“家门口医院”就能看病。加强城乡医保衔接和资金监管，实现基本医疗保险提质扩面。加大大病医疗救助，探索职工门诊统筹医保付费方式。建立食品安全准入机制，健全食品药品行业诚信体系，严厉打击食品药品违法犯罪行为，提升食品药品安全水平。

　　（七）推进社会管理创新。夯实基层基础。深化“天网工程”应用，打造网络化、智能化“数字街区”。逐步推行社区民警专职化，构建立体、联动、网格化治安防控体系，严厉打击各类违法犯罪。完善人民调解网络体系建设，加强特殊人群管理服务与教育。深入基层、深入实际，加强与群众的联系，把矛盾和问题化解在基层。健全常态管理。落实社会管理法治化纲要，争创“全国社会管理示范城市”。加强社区人员和物业管理，提升社区管理水平，实现“社区管理社会化、社会管理社区化”。深化户籍制度改革，促进流动人口基本公共服务均等化。培育社会组织，增强群众自我管理能力。开展第三次全国经济普查。加强电子政务应用建设。建好社会综合治税信息平台。深化国有企业改革，完善国有资产管理。促进社会和谐。推进企业安全生产标准化建设和隐患排查治理系统建设，遏制重特大安全事故。畅通信访渠道，加大调解力度，加大积案老案化解力度，维护改制企业稳定。深入推进“六五”普法和法律援助，做好人口与计划生育工作，保护妇女、儿童、老年人、残疾人合法权益。加强民兵预备役、国防动员和人民防空工作，积极为驻军办实事。促进民族团结进步，依法管理宗教事务。全面做好国家安全、外事侨务、档案管理、机关事务管理、地方志等工作。

　　各位代表！美好幸福的生活要靠劳动去创造，将长沙的美好蓝图变成现实，需要全市人民共同努力。让我们以昂扬进取、只争朝夕的精神状态，聚精会神搞建设，潜心尽力促发展，与全市人民同甘共苦、团结奋斗，共同谱写长沙发展的新篇章！

　　各位代表！人民群众对美好生活的向往就是我们的奋斗目标，全市人民生活更加幸福是我们的不懈追求。让我们在市委的正确领导下，奋发进取，埋头苦干，创造无愧于历史、无愧于时代的光辉业绩，开创长沙更加幸福美好的明天！
